# Arsenic Exposure During Pregnancy: Impacts to Maternal and Offspring Cardiovascular Health

**DOI:** 10.18103/mra.2026.0269

**Published:** 2026-06

**Authors:** Morgan Steiner, Marsha Wills-Karp, Mark Kohr

**Affiliations:** Department of Environmental Health and Engineering, Johns Hopkins Bloomberg School of Public Health, Baltimore, MD, 21205

## Abstract

Cardiovascular disease (CVD) is the leading cause of pregnancy-related deaths, with rates continuing to rise. While older maternal age and preexisting conditions such as obesity, diabetes and hypertension contribute to this trend, they do not fully account for it, suggesting a critical role for environmental factors. A major environmental concern is inorganic arsenic (iAs), which contaminates the food and water supply of over 140 million people worldwide. Exposure to iAs has been linked to a number of chronic diseases including cancer, neurodegenerative disorders and heart disease. During pregnancy, gestational iAs exposure poses a substantial threat to maternal and fetal health. It is linked with a higher risk of maternal hypertension, preeclampsia, and structural heart changes during and after pregnancy. Concurrently, it drives fetal complications including pre-term delivery, congenital heart defects and low birth weight. Furthermore, these early-life exposures predispose offspring to lifelong CVD. Herein, we summarize current evidence regarding the adverse impacts of prenatal iAs exposure on the cardiovascular health of mothers and their offspring. We underscore the need to elucidate the underlying biological mechanisms to inform targeted preventive strategies, therapeutics, and public health policies aimed at combating the rising global maternal mortality.

## 1. Introduction

**Figure 1.**
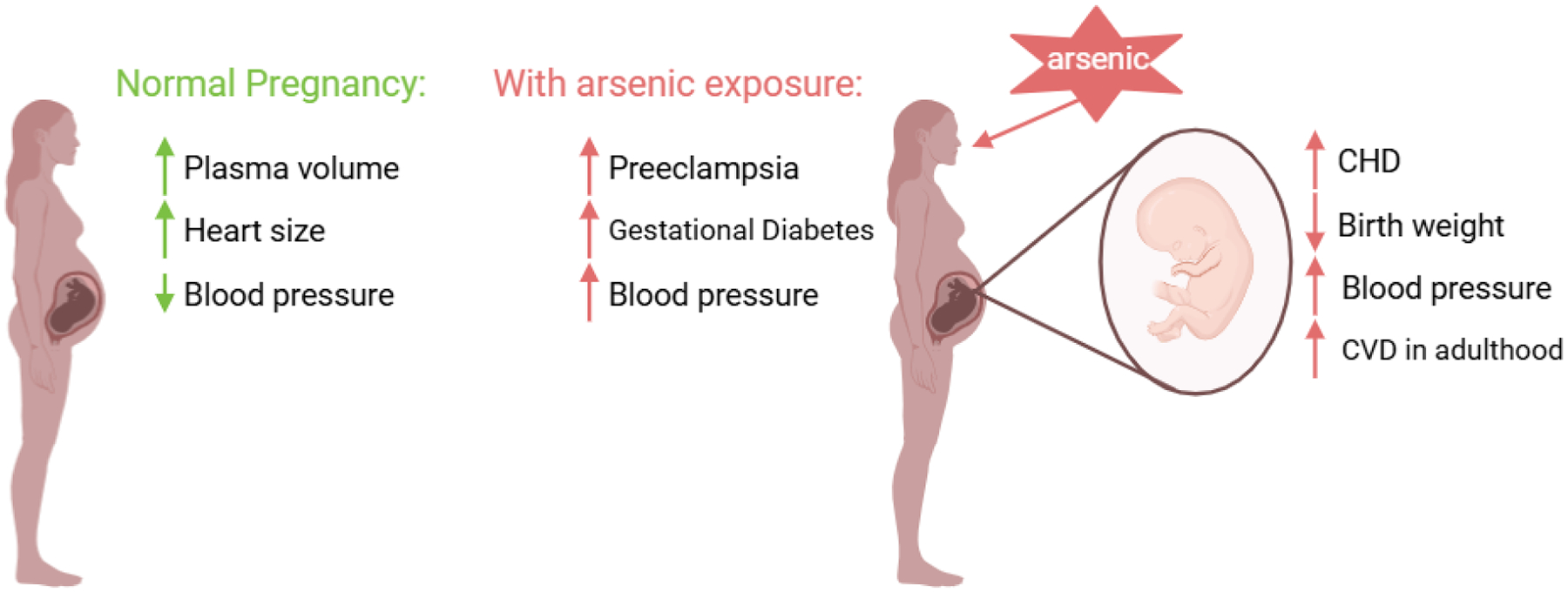
Effect of gestational arsenic exposure on the mother and offspring. Physiological changes typically associated with normal pregnancy are highlighted in green, while detrimental effects of gestational arsenic exposure on the mother and offspring are highlighted in red.

Maternal mortality remains unacceptably high worldwide, with low-income countries experiencing an estimated 346 deaths per 100,000 live births in 2023 compared with about 10 per 100,000 live births in high-income countries^1^. Cardiovascular-related complications are the leading cause of death for women during pregnancy and account for up to one third of maternal deaths worldwide^2–8^. Normal pregnancy is a dynamic process associated with significant physiological adaptations required to meet the increased metabolic demands of the mother and fetus and to ensure adequate placental circulation for fetal development. This process involves hormone-driven expansion of plasma volume and reversible maternal cardiac growth to support fetal development^9–13^. At the onset of pregnancy, rising estrogen levels initiate a signaling cascade that promotes plasma volume expansion^14^. This increase in plasma volume contributes in part to pregnancy-induced cardiac hypertrophy, resulting in greater ventricular muscle mass^15^. Pregnancy-induced heart growth is necessary for maintaining sufficient circulation for proper fetal growth and development and is typically reversible, with postpartum remodeling occurring as early as 1 to 3 months, but sometimes persists for up to one year postpartum in humans^16,17^. These systemic cardiovascular changes impart a substantial stress on the body, but are generally well-tolerated in healthy women (Fig. 1). Yet many women experience cardiovascular complications during or after pregnancy, including gestational hypertension, peripartum cardiomyopathy, and pre-eclampsia, even in the absence of traditional risk factors like genetics or pre-existing conditions (obesity, chronic conditions). Pre-eclampsia, a placenta-mediated disorder marked by high blood pressure and proteinuria after the 20^th^ week of pregnancy^18^, is associated with altered plasma volume expansion^19,20^, which is essential for normal fetal growth. Women who develop pre-eclampsia during pregnancy also have increased risk for developing cardiovascular disease later in life^21,22^, as pre-eclampsia increases the risk of developing peripartum cardiomyopathy^23,24^. Peripartum cardiomyopathy is a life-threatening condition that can develop as early as the final month of pregnancy or up to 5 months postpartum characterized by left ventricular systolic dysfunction and a reduction in cardiac function^25^. Emerging evidence suggests that environmental exposures, including gestational iAs exposure, may contribute to cardiovascular disease in both pregnant women and their offspring (Fig. 1).

### 1.1 ARSENIC EXPOSURE AND ASSESSMENT:

Arsenic is a naturally occurring metalloid found in soil and water, existing in trivalent and pentavalent forms, with trivalent arsenic being the most toxic^26^. Contamination, largely from human activities like mining, use of arsenic-based compounds, and coal burning, is widespread in both drinking water and food supplies^27,28^. Humans are exposed mainly via ingestion, inhalation, and skin contact, with arsenic accumulating in organs such as the liver, kidneys, lungs, bones, skin, hair, and nails, posing serious health risks. Arsenic can be found in combination with organic or inorganic substances and is metabolized via oxidative methylation, glutathione conjugation^29,30^, or through a more recently identified reductive methylation pathway^31^. Arsenic methyltransferase is responsible for methylating and detoxifying arsenic into its methylated metabolites, monomethylarsonic acid and dimethylarsinic acid^10^. Measurements of arsenic, its methylated metabolites, and total arsenic in a variety of tissues (blood, urine, toenails) are considered reliable biomarkers of exposure. Over 140 million people worldwide are exposed to arsenic in drinking water at levels that exceed the drinking water guideline of 10 μg/L set by the World Health Organization. India and Bangladesh are most affected, with groundwater levels of 100–300 μg/L^13^, but high arsenic is also reported in the United States, Chile, China, Taiwan and Mexico. As such, arsenic ranks second on the World Health Organization’s list of chemicals of public health concern^32^.

### 1.2 ENVIRONMENTAL ARSENIC EXPOSURE AND CARDIOVASCULAR DISEASE RISK

Population studies link arsenic exposure to a variety of diseases including cancer, neurodegenerative disease, and various forms of cardiovascular disease^33–36^. In particular, epidemiologic studies in areas of the world with high levels of arsenic that include Bangladesh, Taiwan, Italy and parts of the United States, associate arsenic exposure with hypertension^37–39^, vascular dysfunction^39,40^, atherosclerosis^41,42^, coronary heart disease^36,43,44^, stroke^36,43,45,46^, and higher cardiovascular mortality^36,40,43,43,47^. Low to moderate exposure to arsenic in drinking water (100 μg/L or less) is also common in many parts of the world, and is similarly associated with both fatal and non-fatal forms of cardiovascular disease^43^, and alterations to heart structure^48^. Moreover, cardiovascular risk appears to be dose-dependent, with affects observed even at low-to-moderate levels of arsenic exposure (<100 μg/L)^36^. Similar cardiovascular changes have been noted in animal studies, with arsenic exposure increasing blood pressure^49,50^, and inducing QT prolongation and bradycardia (slow heart rate)^51,52^. Studies have also noted sex-specific pathological effects of acute and chronic arsenic exposure on the structure and ischemic susceptibility of the heart in mouse models, with males showing greater susceptibility to ischemic injury than females following arsenic exposure^49,50,53^. Collectively, prior studies in humans and animals support arsenic exposure as a primary driver of cardiovascular disease.

## 2. Impact of Gestational Arsenic Exposure on Maternal Cardiovascular Health

### 2.1 IMPACT OF ARSENIC ON MATERNAL CARDIOVASCULAR HEALTH DURING PREGNANCY

Exposure to arsenic during pregnancy poses significant health risks to mothers and developing fetuses. Even relatively low concentrations in drinking water (20–50 μg/L) have been associated with increased maternal systolic blood pressure during pregnancy and postpartum^54,55^. For instance, Farzan et al. showed in the New Hampshire birth cohort study that higher urinary arsenic levels in pregnant women were linked to greater increases in systemic blood pressure and pulse pressure throughout gestation^55^. Beyond gestational hypertension, arsenic exposure has been linked to pre-eclampsia^56–58^. This condition, along with other pregnancy-related cardiovascular complications, not only threatens maternal and fetal health at the time (acutely or during pregnancy), but also elevates the mother’s long-term risk of developing cardiovascular disease^18,22^.

**Mechanistically**, arsenic may increase cardiovascular complications in pregnancy by impairing plasma volume expansion, which is linked to preeclampsia^19^. A previous study also noted an association with higher postpartum blood pressure^59^, suggesting arsenic toxicity may persist into the postpartum period. However, assessing the effects of arsenic remains challenging in pregnancy due to exposure variability and physiological factors like plasma volume changes.

### 2.2 EFFECT OF GESTATIONAL ARSENIC EXPOSURE ON MATERNAL CARDIOVASCULAR HEALTH IN ANIMAL MODELS

To investigate the impact of arsenic exposure on pregnancy, researchers have utilized mouse models of gestational exposure. Taube *et al*. found that exposing pregnant mice to iAs in their drinking water blunted normal maternal heart enlargement in late pregnancy when compared to non-exposed pregnant controls^60^. The suppression of cardiac hypertrophy was associated with reduced cardiomyocyte cross-sectional area and decreased transcriptional levels of *Nppa* (atrial natriuretic peptide)^60^ and protein levels of protein kinase B (Akt)^60^, key drivers of pregnancy-induced heart growth^61,62,63^. Additionally, pregnant mice exposed to iAs showed a significant increase in cardiac output compared to non-exposed pregnant mice, and exposed non-pregnant females, concomitant with increases in stroke volume. As iAs-induced functional cardiac changes were absent in non-pregnant exposed females, the investigators concluded that pregnancy is a state of particular vulnerability to iAs exposure. Since plasma volume expansion and subsequent heart growth are essential to ensure proper maternal and fetal circulation, the significant abrogation of maternal cardiac hypertrophy could lead to insufficient blood flow in maternal and fetal tissues and complications for both the mother and fetus during pregnancy.

Notably, at postpartum timepoints, Taube *et al*. found that gestationally iAs-exposed dam hearts were larger compared to non-exposed postpartum controls^64^, two weeks after the cessation of arsenic exposure. Postpartum cardiac dysfunction was also observed at the level of the cardiomyocyte, as calcium-handling and cardiomyocyte contraction were significantly impaired in gestationally-exposed dams as compared to non-exposed controls^64^. Arsenic-induced cardiac enlargement could lead to long-term maternal cardiovascular complications.

In addition to changes in heart size and function, arsenic exposure during pregnancy has been shown to disrupt placental vasculogenesis and placentation in pregnant mice, through epigenetic mechanisms that alter the expression of platelet-derived growth factor subunit B (*Pdgfb*)^65,66^. Mechanistically, iAs alters *Pdgfb expression by* inhibiting Ten-eleven translocation (TET) enzyme activity and a-ketoglutarate content. Supplementation with a-KG was shown to ameliorate As-induced alteration of *Pdgfb* epigenetic programming, concomitant with improvement of iAs-induced aberrant placental angiogenesis and fetal growth restriction. Defective placental vascular remodeling can contribute to maternal hypertension and pre-eclampsia as well as disruption of fetal growth and development resulting in lifelong health consequences.

Pregnancy-induced cardiovascular changes are governed largely by sex-hormones such as estradiol, and progesterone. iAs has been shown to act as an endocrine disruptor through reducing estrogen- and progesterone receptor–dependent gene transcription^67–69^. Consistent with the estrogen-disrupting effects of arsenic, Taube et al. found that gestational arsenic exposure reduced ERα and progesterone membrane receptor (*Pgrmc1, Pgrmc2*) expression in the maternal heart during late pregnancy^60^. As these hormones are critical regulators of the physiological changes in the pregnant women that are required to maintain a pregnancy, arsenic disruption of these hormonal pathways can have widespread impacts on both the health of the mother and the fetus. For example, estrogen and progesterone exert strict control over the expression and activation of the key drivers of maternal heart growth mentioned above (Akt, Nppa). Arsenic may also disrupt cardiovascular adaptations during pregnancy through its ability to promote oxidative stress and endothelial dysfunction^70^. The pro-oxidative stress effect of iAs may be compounded in pregnancy by its ability to reduce estrogen which has been shown to function as an anti-oxidant^71^. Collectively, these findings in animal models suggest that gestational arsenic exposure disrupts maternal cardiac growth and function during and after pregnancy, and induces placental dysfunction, with potential effects persisting beyond the arsenic exposure period^60,61,64,65^. As differences exist in pregnancy and the metabolism of iAs between humans and mice, further studies in humans are clearly warranted to better understand the adverse impacts of iAs exposure on maternal cardiovascular health.

## 3. Impact of Gestational Arsenic Exposure on Offspring Health

### 3.1 GESTATIONAL ARSENIC EXPOSURE AND ADVERSE FETAL OUTCOMES

While adverse maternal outcomes associated with gestational arsenic exposure are not well understood, extensive evidence links gestational arsenic exposure to adverse birth outcomes, as arsenic readily crosses the placenta^72,73^, leading to direct fetal exposure. Moderate to high exposure (exceeding 555 μg/L normalized to creatinine) increases the risk of stillbirth^74^. Infant mortality risk also increases with arsenic exposure greater than 1 μg/L in the population^75^. Maternal urinary arsenic levels are also associated with low birth weight (<5.5 lbs. according to WHO^75^)^76–78^, shorter gestation time (pre-term birth), and reduced newborn length^79–81^, particularly in females and during the third trimester^79,80,82–86^. These findings suggest that the current maximum containment level for arsenic (10 μg/L) may not be sufficient to protect against adverse birth outcomes^87^.

Furthermore, evidence indicates a strong association between gestational arsenic exposure and cardiovascular alterations, particularly congenital heart defects (CHD), the most common type of birth defect and a leading cause of neonatal mortality^35^. Epidemiological studies in countries that include France, Hungary, China, and Denmark report increased CHD risk with prenatal arsenic exposure, even at low concentrations (0.5–0.9 μg/L)^76–79^. While these studies were primarily associated with maternal blood or urinary arsenic measures, a separate study conducted in China found an association between increased arsenic levels in the hair of pregnant women and a higher risk of coronary heart disease in their offspring^92^. Gestational exposure has also been linked to congenital anomalies, especially heart defects, among women exposed to >10 μg/L arsenic in drinking water^71,79,80,82,83,88^. Notably, some studies found this association in female but not male offspring^92^, highlighting the need for further research into sex-specific effects and underlying mechanisms.

Similar associations between prenatal arsenic and cardiac malformations have also been reported in animal models. Offspring of mice exposed to arsenic-contaminated water before and during pregnancy show increased atrial septal defects and other heart abnormalities^93^. higher heart weight–to–body weight ratios^60^, and enlarged left ventricular diameter in late gestation^60^. In rats, exposure to 5 μg/L arsenic impaired growth and development^94^. Together with epidemiological findings, these data strengthen the association between gestational arsenic exposure and congenital heart defects in offspring.

### 3.2 MECHANISMS OF ARSENIC-INDUCED TOXICITY IN CARDIAC DEVELOPMENT

Although in vivo mechanistic studies in humans are limited, both in vitro and animal models have been useful in determining the cellular targets of arsenic during development. In chick embryos and coronary/epicardial progenitor cells, arsenic and its metabolite MMA disrupted epithelial-to-mesenchymal transition via interference with TGF-β/Smad signaling^95–97^. In zebrafish, arsenic impaired formation of the ventricle, delayed cardiac looping, reduced heart rate, and altered chamber structure, partly through downregulation of Dvr1^52,98^. In mammals, Winterbottom et al. found that gestational arsenic exposure decreased placental expression of *PRDM6*, a gene associated with congenital heart defects^99^. Furthermore, in rats, arsenic exposure during periconception increased the risk of offspring septal defects, while folic acid supplementation provided sex-specific protection by limiting histone H3K9 acetylation and reducing Mef2C expression^100^. Despite these findings, further research is needed to clarify sex-specific susceptibility to gestational arsenic exposure.

Another potential mechanism of arsenic-induced developmental cardiotoxicity involves disruption of one-carbon metabolism, which generates intermediates such as S-adenosylmethionine (SAM) and glutathione required for arsenic methylation and detoxification^101^, as SAM is the methyl donor necessary for the production of arsenic metabolites, MMA and DMA. Although this pathway is upregulated during pregnancy to support fetal growth^102^, arsenic exposure may impair one-carbon metabolism by inducing nutritional deficiencies, including low vitamin B12 and elevated homocysteine—markers of reduced methylation capacity. These alterations are associated with higher urinary and cord serum arsenic and MMA levels, as well as reduced birth and placental weights^79,103^. Moreover, while elevated MMA is a known cardiovascular risk factor in adults^104,105^, its specific effects on maternal and fetal health remain poorly understood.

### 3.3 GESTATIONAL ARSENIC EXPOSURE AND CARDIOVASCULAR DISEASE RISK IN CHILDHOOD AND ADULTHOOD

Some of the earliest evidence linking *in utero* arsenic exposure to cardiovascular disease comes from autopsies of children in Antofagasta, Chile, where high drinking-water contamination occurred from 1958–1970^106,107^. Exposed children showed widespread vascular lesions, including fatal acute myocardial infarction. Ecological studies later found that men born during peak contamination had nearly triple the rate of acute myocardial infarction mortality compared to the general Chilean population^108^. Additional research associates gestational arsenic exposure with long-term risks such as arterial plaque formation^109–111^, peripheral vascular disease^112^, arterial thickening and vascular lesions^113^.

Moreover, extensive evidence links gestational arsenic exposure to elevated childhood blood pressure. Studies in Mexico and Bangladesh associate early-life exposure with higher blood pressure, cardiac hypertrophy, carotid intima media thickness and plasma asymmetric dimethylarginine, a marker of oxidative stress^114–116^. In Bangladesh, each 1 mg/L increase in maternal urinary arsenic was associated with 3.69 mmHg and 2.91 mmHg increases in systolic and diastolic BP, respectively, at 4.5 years of age^114^. Similar trends appear in Chinese cohorts^114^ and animal models, though rat studies suggest a sex-specific vulnerability in females^118^. Because childhood hypertension often tracks into adulthood, gestational arsenic exposure may contribute to long-term cardiovascular risk.

Mechanistically, arsenic may promote long-term cardiovascular disease by acting as an epigenetic modifier, altering fetal DNA methylation in ways that persist into adulthood^101,119–121^. Arsenic exposure has been shown to change methylation and expression of genes linked to cardiovascular disease^122^, and human studies associate gestational arsenic exposure with adult peripheral vascular disease^123^, and increased myocardial infarction risk^124–126^. However, inconsistent findings in animal models, such as the study conducted in mice by Rychlik *et al*.^120^ which did not observe enhanced susceptibility myocardial ischemic injury, suggest that arsenic effects may depend on the timing of exposure. Impaired growth hormone/insulin-like growth factor signaling observed in zebrafish embryos further implicates developmental pathways relevant to cardiac dysfunction^127,128^.

Overall, the mechanisms linking prenatal arsenic exposure to adult cardiovascular disease remain incompletely understood and require further study.

## 4. Conclusions

In summary, strong evidence links gestational arsenic exposure to adverse cardiovascular outcomes in both mothers and offspring during and after pregnancy. As maternal mortality rises globally and congenital heart defects remain the top cause of neonatal mortality, understanding how arsenic disrupts the normal physiology of pregnancy is essential to identifying actionable points for intervention. Strengthening public health protections, including stricter regulatory limits in food and water, improved exposure monitoring, and development of targeted therapies, may help reduce the cardiovascular burden associated with arsenic exposure, especially in vulnerable populations.
